# Large conformational changes of a highly dynamic pre-protein binding domain in SecA

**DOI:** 10.1038/s42003-018-0133-4

**Published:** 2018-09-03

**Authors:** Isabel Ernst, Maximilian Haase, Stefan Ernst, Shuguang Yuan, Andreas Kuhn, Sebastian Leptihn

**Affiliations:** 1 University of Hohenheim, Institute for Microbiology and Molecular Biology, Garbenstr. 30, 70599 Stuttgart, Germany; 2 Ecole Polytechnique Fédérale de Lausanne (EPFL), Laboratory of Physical Chemistry of Polymers and Membranes, CH-1015 Lausanne, Switzerland; 3 Zhejiang University-Edinburgh University (ZJU-UoE) Institute, Zhejiang University, 718 East Haizhou Road, Haining, Zhejiang 314400 China

## Abstract

SecA is an essential molecular motor for the translocation of proteins across the membrane via the bacterial Sec secretion system. While the Sec system is found in all cells from archaea to multicellular eukaryotes, the SecA protein is mainly found in bacteria. The mechanism of how the motor protein works on a molecular level is still under dispute but it is well established that SecA binds ATP and uses its hydrolysis for the translocation of substrates. In this work, we addressed the question of which conformational changes the protein might undergo during protein translocation. To this end, we investigated the molecular movements of SecA in the absence or the presence of ATP using single-molecule FRET measurements and in silico normal mode analyses. Our results demonstrate that the pre-protein binding domain of SecA is highly dynamic in the absence of the nucleotide and moves towards the helical wing domain in an ATP-bound state.

## Abstract (web summary)

Isabel Ernst et al. use single-molecule FRET measurements and in silico analyses to show the conformational changes of bacterial SecA. They show that the Preprotein Binding Domain is highly dynamic in the absence of ATP and moves toward the Helical Wing Domain when ATP is bound into the “wide open” position.

## Introduction

**Fig. 1:**
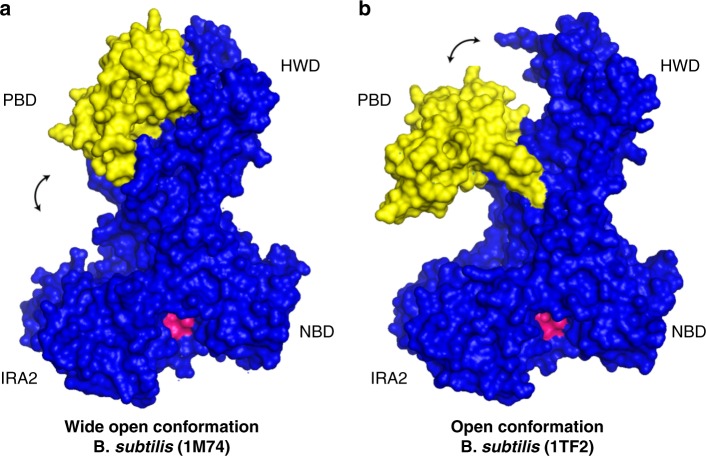
The position of the pre-protein binding domain (PBD) is not a common denominator in known SecA structures. Atomic model of SecA proteins from *B. subtilis* in the (**a**) so-called wide open conformation (1M74) and (**b**) in the open conformation (1TF2), respectively. The two SecA crystal structures depicted here were both obtained in the presence of adenosine diphosphate (ADP) and differ largely by the position of the PBD (yellow). The ADP binding cleft is coloured in pink. The double-headed arrow highlights the proposed mobility of the PBD

In bacteria, about 30% of newly synthesised proteins are exported outside the cytoplasm^1^. Sophisticated export machineries facilitate the transport of these proteins across the cytoplasmic membrane. The most important and universally conserved membrane complex that allows the translocation of proteins across the membrane is the Sec protein complex, with the central translocon pore within SecY in bacteria and of Sec61α in eukaryotes. In bacteria, an essential motor protein called SecA, absent in eukarya with the exception of chloroplasts, associates with the membrane-embedded Sec translocon and facilitates substrate transport, using the energy provided by the hydrolysis of adenosine triphosphate (ATP)^2^. The nucleotide is bound at an interface of the nucleotide-binding domain (NBD or NBD1, as found in the literature) and the intramolecular regulator of ATPase2 (IRA2 or NBD2) (Fig. 1). The NBD exhibits a stem structure that connects it to the pre-protein binding domain (PBD), which is important for the protein substrate recognition. The C-terminal domain (C-domain) is connected to IRA2 and composed of the helical scaffold domain, a connector of all domains within SecA. The C-domain contains the IRA1 domain (also known as the two-helix-finger), the helical wing domain (HWD) and the C-terminal tail^1^. The mechanism of how the motor protein works on a mechanistic level is still under dispute^3-5^. In the current model, the translocating protein chain is dragged by the two-helix-finger into the SecY translocon pore^4,6^. Over the years, many different SecA structures have been obtained by crystallography using proteins from various bacteria. Among those, three distinct positions of the PBD have been observed with one being closer to the HWD of the protein the “wide open conformation”, as is in case of SecA of *Bacillus subtilis* (1M74), the second where the PBD is closely associated with the IRA2 domain as is in the case (3DIN) of the thermophile *Thermotoga maritima* called “the closed conformation”, and a third one, the “open conformation” in which the PBD is positioned in between the two mentioned domains (1TF2) of *Bacillus subtilis* or (2FSF) of *Escherichia coli* SecA^7-9^. The closed conformation was only observed when SecA was bound to SecYEG^7^ whereas in solution SecA adopts the open and the wide open state^2^. Figure 1 shows the two structures from *B. subtilis* which we used for our study in order to address the question of conformational changes within SecA in solution and potential movements of the PBD within the protein. Both the open conformation of the SecA molecule (Osborne et al.^9^, PDB: 1TF2) and the wide open conformation of SecA from *B. subtilis* (Hunt et al.^8^, PDB: 1M74) had been crystallised in the presence of adenosine diphosphate (ADP) (Fig. 1). Using this information, we attempted to understand the dynamic movements of the PBD within the protein that occur during translocation, which involves the binding and the hydrolysis of ATP. ATP, however, is bound at the interface between IRA2 and the NBD, quasi-opposite from the PBD. Therefore, it was unclear whether the binding of a nucleotide influences the position of the PBD in SecA.

In this study, we demonstrate using single-molecule Förster resonance energy transfer (smFRET) that the binding of ATP has a clear effect on the conformation of SecA. We find that when ATP is present, the PBD is positioned close to the HWD, while in the absence of the nucleotide the PBD is further apart from the HWD. In addition, PBD shows to be a highly dynamic domain which, in the absence of ATP, can be found in several positions but with a higher probability where PBD is close to the HWD. We also analysed the conformational change of SecA using in silico experiments (normal mode analysis (NMA)), which confirms our findings.

## Results

### ATP binding causes a conformational change in SecA

To understand the movements of SecA domains relative to each other during the translocation process of substrate proteins, which involves the hydrolysis of ATP, we measured the distance between the PBD and the HWD. In a SecA derivative where all 4 cysteine codons had been substituted with serine codons, we constructed a double cysteine mutant with one substitution to cysteine in the PBD (K329C) and the other substitution, P704C, in the HWD. The residues were chosen as they are surface exposed in both domains. After expression in *Escherichia coli* BL21 (DE3) and purification to homogeneity, the protein was labelled stochastically with an equimolar ratio of donor and acceptor dye. As no FRET signals can be observed when only one of the residues is modified by a dye, or two donor/acceptor dyes are bound by the two cysteines in SecA, FRET signals can only be recorded from proteins that were modified with one FRET pair dye molecule each. Also, as we employ single-molecule fluorescence, the positioning of either dye is irrelevant as bound dye molecules will only allow to generate FRET signals regardless of their position (e.g., 329C-Atto647N and 704C-Atto520 *OR:* 329C-Atto520 and 704C-Atto647N).

**Fig. 2:**
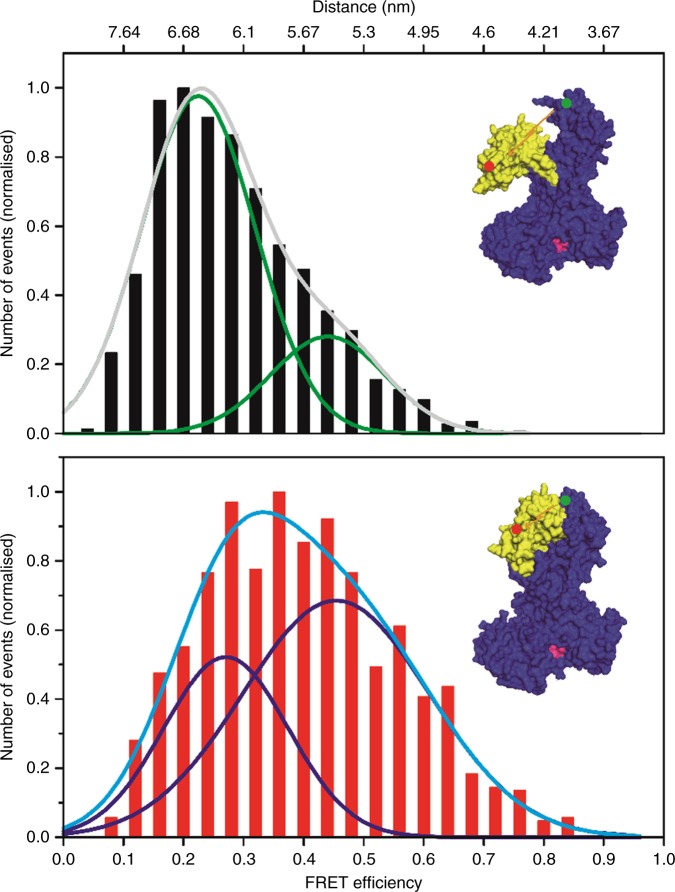
The pre-protein binding domain (PBD) moves closer to the helical wing domain (HWD) upon adenosine triphosphate (ATP) binding. Histograms of the single-molecule Förster resonance energy transfer (smFRET) data obtained with SecA 329C-704C, in the absence (top panel) and the presence of ATP (bottom panel). The data can be fitted best with two Gaussian distributions. The distance between the two fluorophores in the PBD and in the HWD decreases when ATP is present from 6.5 nm to 5.5 nm, indicating that the PBD moves towards the HWD. Insets show the position of the fluorescent labels in SecA. To illustrate the movement of the PBD, the atomic SecA structures 1TF2 (top) and 1M74 (bottom) are shown. The distance between the PBD at residue 329 (red dot) and the HWD at 704 (green dot) is highlighted with arrows and is 5.6 nm in the *B. subtilis* open structure (1TF2) and 4.5 nm in the wide open structure (1M74) between the corresponding residues (D309 and V658)

Using the single-molecule approach fluorescence correlation spectroscopy (FCS), the concentration of SecA is well below the dimer/monomer dissociation constant of SecA^9^ and thus only single SecA monomers were measured. Therefore, signals that might originate from inter-subunit FRET are avoided. This was furthermore confirmed by the observation that only fluorescence intensities were obtained that were consistent with monomeric SecA corroborated by the diffusion coefficient. We therefore only measured FRET occurring between the two dye molecules covalently bound to residues 329 and 704 within one SecA protein. Signals were selected using an automated search algorithm (see Methods). From the FRET signals the so-called proximity factors can be derived, which in turn can be used to establish the distances between two fluorophores. Of altogether 1025 events in the absence of ATP, we derived distance values between the dye molecules that were plotted against their frequency. The resulting histogram was fitted with two Gaussian distribution curves which exhibited one maximum at approximately 6.5 nm and a second minor curve with a maximum at approximately 5.5 nm (Fig. 2, top panel). Once ATP was added, the peak shifted to a higher FRET efficiency, indicating a reduction of the distance between the two fluorophores. Here, the 1029 events collected in the presence of the nucleotide were analysed and again plotted with their distances against the frequency. The distribution was best fitted with two Gauss curves with the major having a maximum at approximately 5.5 nm and a second minor curve with a maximum at around 6.3 nm, indicating that the binding of ATP resulted in a conformational change within SecA moving the PBD closer to the HWD (Fig. 2, bottom panel).

### PBD moves away from IRA2 and towards HWD upon ATP binding

An intramolecular conformational change could also be explained by the movement of the HWD towards the PBD. However, the various crystal structures show that the distance variation between IRA2 and HWD is small and an inspection of the respective residues 472 and 704 in the two crystal structures shows about 0.5 nm closer distance in open structure. Nonetheless, we measured FRET between residue 329 in the PBD and residue 472 in the IRA2 with the appropriate SecA mutant of K329C/A472C. If the PBD moved towards the HWD upon the binding of ATP, then the distance between PBD and IRA2 should become larger when the protein is in its nucleotide-bound state. This hypothesis could be confirmed by the data obtained in our measurements.

**Fig. 3:**
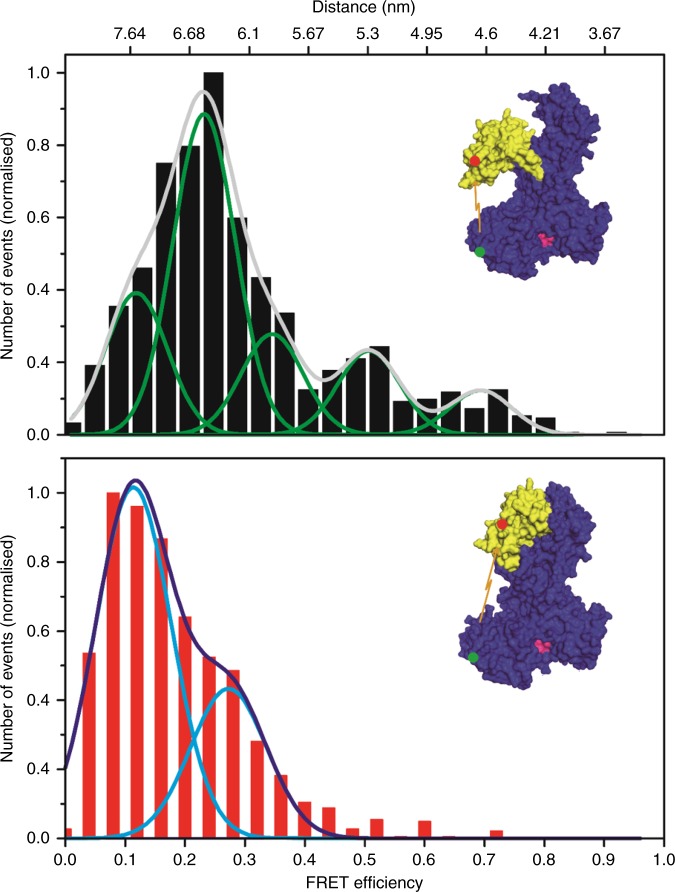
Upon adenosine triphosphate (ATP) binding, the pre-protein binding domain (PBD) moves away from the IRA2 domain. Histogram of the single-molecule Förster resonance energy transfer (smFRET) data obtained with SecA 329C–472C, in the absence (top) and the presence of ATP (bottom). While the data in the presence of ATP can be fitted with a bimodal Gaussian distribution, the data in the absence of nucleotide is best fitted with a multimodal function. The distance between the two fluorophores in the PBD and the IRA2 domain increases when ATP is present from 6.5 nm to 7.5 nm, indicating that the PDB moves towards the helical wing domain (HWD). Insets show the position of the fluorescent labels in SecA. To illustrate the movement of the PBD, the atomic SecA structures 1TF2 (top) and 1M74 (bottom) are shown. The distance between the PBD at residue 329 (red dot) and the IRA2 domain at 472 (green dot) is highlighted with arrows and is 5.1 nm in the *B. subtilis* open structure (1TF2) and 6.1 nm in the wide open structure (1M74) between the corresponding residues (D309 and K452)

We analysed a total of 1062 events in the presence of ATP and 1021 in the absence of the nucleotide. In the presence of ATP, a distribution was observed which was fitted best with two Gaussian functions, resulting in one major curve with a maximum at approximately 7.6 nm and a second minor one with a maximum at 6.2 nm between the PBD and the IRA2 (Fig. 3, bottom panel). However, this was different when the distance between PBD and IRA2 was measured in the absence of the nucleotide which could be best fitted with a penta-modal distribution model (Fig. 3, top panel). Here, the major of the five maxima exhibited a similar distance as the minor peak we had observed in the presence of ATP (6.5 nm).

We also observed a minor peak at 7.5 nm, while the third, fourth and fifth minor maxima were determined to exhibit distances of ~5.9, ~5.3 and ~4.6 nm, respectively. This frequency distribution of events indicated that in the absence of ATP, the PBD is rather dynamic and molecules can exhibit a variety of conformations. It appears that the PBD is more often closer to the HWD (reflected by a higher frequency of events) than the conformations in which the PBD is closer to the IRA2, which is similar to the crystal structure of *B. subtilis* 1TF2 (Fig. 1a). In the absence of ATP, the events that were observed indicate the domain not being locked in an either/or position, i.e., close to the HWD or the IRA2, respectively. Since it is likely that SecA readily hydrolyses the bound ATP, we also measured the distance between PBD and IRA2 in the presence of the non-hydrolysable adenylyl-imidodiphosphate (AMP-PNP) and analysed 516 collected events (Supplementary Fig. 1). The distances we observed after the addition of AMP-PNP suggest that the binding of AMP-PNP has a similar effect as that of ATP and the PBD is locked in the wide open position (Fig. 3, bottom panel).

### Normal mode analysis confirms single-molecule FRET results

**Fig. 4:**
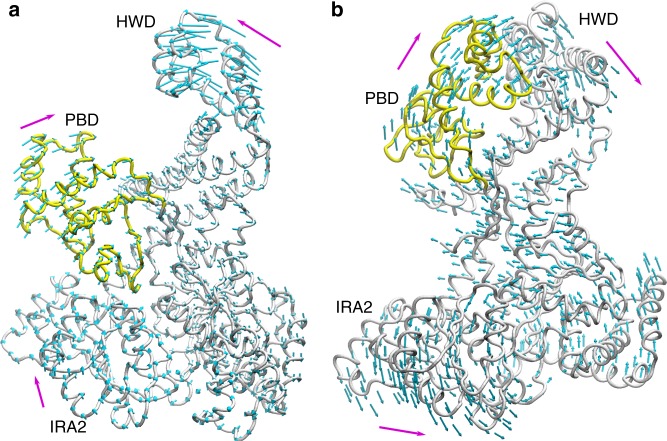
Structural fluctuations of domains in SecA proteins. Normal mode analyses (NMA) of both the open (**a**) and the wide open (**b**) conformations of SecA. The figure shows the intrinsic movements of each domain. The arrow lengths and colour are correlated to their fluctuations. In both structures the pre-protein binding domain (PBD; yellow) moves towards the helical wing domain (HWD). The major difference between the two conformations regarding their intrinsic movements refers to the IRA2 and HWD which move towards the PBD in the open conformation (**a**), or away from the PBD in the wide open state of SecA (**b**)

NMA, which is used to investigate large conformational dynamics of a protein, was employed to characterise the molecular movements of the domains within SecA. The analyses demonstrate that the PBD has a pronounced tendency to move towards the HWD in a nucleotide-bound state, which confirms our smFRET data. In both structures, which represent the open conformation (1TF2) and the wide open conformation (1M74) of SecA, when bound to a nucleotide (ADP) the PBD moves towards the HWD (Fig. 4, supplementary movie 1). In the SecA structure which Zimmer et al.^7^ describe as being in “an intermediate state during ATP hydrolysis” (3DIN), both the HWD and the IRA2 move towards the PBD, with the latter displaying a movement towards the HWD (Fig. 4a). The picture of SecA in a ADP-bound state (1M74) is different^8^; here, the PDB is close to the HWD but still moves towards the HWD which in turn shows a tendency to move towards the NBD (Fig. 4b, supplementary movie 2). Similarly, the IRA2 domain moves towards the NBD. Whether this could represent a conformation preceding the release of the diphosphate nucleotide after hydrolysis remains to be investigated.

## Discussion

The translocation of proteins across the bacterial inner membrane is catalysed by the Sec translocase and YidC insertase^10^ that are conserved across all domains of life^1,11^. In bacteria, the essential protein SecA is an additional component of the Sec translocase that first binds the pre-protein and then moves the polypeptide chain into the translocation channel of SecY that moves the protein in a linear fashion to the periplasmic face of the membrane. The SecA motor protein is not only involved in the translocation of many periplasmic and outer membrane proteins across the cytoplasmic membrane, but is also required for the insertion of some inner membrane proteins that have a large periplasmic domain. To fulfil its function, SecA uses the energy from the hydrolysis of ATP. Molecular motors are fascinating nano-machines that use the energy provided by the hydrolysis of tri-phosphate nucleotides to perform mechanical work^12,13^. ATP is bound at the interface of two domains, the NBD and IRA2 domain of SecA (Fig. 1a). The binding of the pre-protein and the hydrolysis of ATP results in large conformational changes that rearrange the topology of the different SecA domains. In our study, we addressed the question of how the SecA domains, in particular the PBD, move relative to the HWD and the IRA2 domains in the absence or the presence of ATP.

**Fig. 5:**
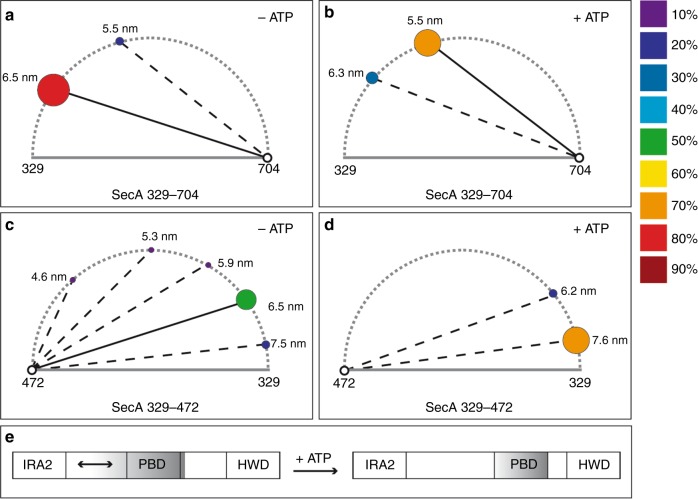
Model of the change in Förster resonance energy transfer (FRET) efficiencies of SecA in the presence or absence of adenosine triphosphate (ATP). FRET efficiencies were calculated in distances (numbers next to circles in nm). The relative probabilities of the distances are displayed colour-coded (right panel), with darker colours (purple/blue/cyan) representing lower probability. Brighter colours (yellow/orange/red) represent higher probabilities. The sizes of the circles are represented relative to the frequency of events, correlating to the integral of their Gauss curves. The addition of ATP to SecA 329–704 results in a conformational change where the pre-protein binding domain (PBD) moves closer to helical wing domain (HWD) (**a**, **b**). In the absence of ATP, the FRET data for SecA 329–427 indicate the existence of several different conformations with one major conformation where IRA2 idles to approach PBD (c). The movement of the PBD to HWD in SecA 329–427 is more prominent in the presence of ATP (**d**). A domain representation (**e**) summarises our interpretation

Our study revealed two observations. First, the results of our FRET analysis allows the conclusion that in a nucleotide-bound state, the PBD moves towards the HWD. Figure 5 summarises this in a model which displays the relative occurrence of distances and the changes between each other. The second observation revealed a dynamic movement of the PBD in the absence of the nucleotide and that PBD varied in its distance to IRA2 (Fig. 3, top panel). This observation might explain why so many different crystal structures of SecA proteins have been obtained. While the overall architecture is highly conserved from thermophiles to other microbes including *E. coli*, the position of the PBD varies in the nucleotide-free state when analysed by nuclear magnetic resonance^14^. Our data indicate that the PBD displays dynamic movements in which during crystallisation a particular conformation might be stabilised by certain conditions, thus leading to the variety of SecA structures. When the FRET distances we obtained between the SecA residues 329 and 704 of *E. coli* are compared with the distances of the homologous residues in the two published structures of *B. subtilis*, we found a similar effect: for both, the distance was changed by about 1 nm (Fig. 2). The observation that in the absence of ATP, SecA does not have a single, rigid conformation is in agreement with the view that proteins exist as sets of molecules with related conformations, an ensemble. While at first glance it seems surprising that the addition of ATP (and even more so of AMP-PNP) to SecA results in a structural rearrangement in a domain that is in a quasi-opposite orientation of the nucleotide-binding site, the principle of allostery explains this observation. This allosteric perturbation, where the binding of an effector at one site of the protein results in a change at a second location, shifts the distribution of the entire population of protein conformations towards one main state^15,16^. We also observe this conformation in the absence of the effector (ATP), although not as the predominant one, which confirms the hypothesis that allosteric structural perturbations do not create new conformations but solely change their relative distribution within the ensemble^15^. Also, as the protein is an ATP-driven motor, the transduction of energy by the hydrolysis should allow the overall movement of the protein to facilitate substrate translocation across the membrane through the Sec complex. This movement across the domains of SecA is visualised by the in silico analyses using the NMA. NMA is an efficient method to sample the large intrinsic movement of a protein^17,18^. NMA shows that in the absence of ATP (Fig. 4a), PBD and IRA2 move towards the same direction, resulting in PBD and IRA2 being close to each other. In contrast, in the presence of ATP (Fig. 4b), PBD and IRA2 move to the opposite directions, resulting in a larger distance between each other. Our observations could also explain how protein substrates of various sizes can be bound by SecA. A size adjustment of a molecular clamp is necessary to tightly hold a protein substrate before and during translocation. Similar to the pipe fitting wrench, a plumber’s tool, SecA can adjust its clamp size at distinct levels as shown by our data where we observed several distinct distances between the PBD and the IRA2 domain (Fig. 3, top panel). Since the distance between the PBD and HWD does not show the reciprocal mobility (Fig. 2, top panel) this size adjustment might result from movements of the IRA2 domain. A movement of IRA2 towards PBD is also suggested by the results of our NMA study (Fig. 4a). While the movement of the PBD in the presence and the absence of nucleotide is a fascinating observation as ATP is not bound by nearby residues, the question of how a pre-protein is bound and how it affects the conformation remains to be investigated. In our study we measured the molecular movements in the SecA monomer, while under in vivo conditions SecA exists in both monomeric and dimeric forms. Although in the cytosol, SecA is dimeric^19,20^, it is discussed that SecA functions as a dimer or monomer during the translocation of substrates across the membrane, while bound to SecYEG^2^. Therefore, the movements we observed might also occur in the SecA dimer. Again, single-molecule measurements may be useful to address these questions on a molecular level.

## Methods

### Expression, purification and fluorescent labelling of SecA

The pIMBB258 plasmid used is a derivate of pZ52, in which all 4 cysteine codons had been substituted with serine codons and the codons for the first five amino acids of SecA from *E. coli* had been deleted and replaced by a hexa-histidine tag^21^. Therefore, we re-introduced the first codons using a QuickChange protocol according to the manufacturer's description. Also using site-directed mutagenesis, two codons were changed to code for cysteines, resulting in proteins with two cysteines, either in position 329 and 704 or 329 and 472 and an N-terminal hexa-histidine tag after translation. The SecA mutants were expressed in BL21 (DE3) cells in LB medium containing 100 µg mL^−1^ ampicillin at 37 °C to mid-log phase and induced with 0.5 mM IPTG for 2 h. Cells were then resuspended in 50 mM Tris-HCl pH 8, 50 mM KCl, 5 mM MgCl_2_, 2 mM dithiothreitol (DTT) pH 8, and purified by nickel affinity chromatography (Ni-NTA, Qiagen, Hilden, Germany), followed by size exclusion chromatography on Superdex 200 (16/60; GE Health Care) that removed DTT. SecA fractions (0.2 mg mL^−1^) in 50 mM Tris-HCl pH 8, 50 mM KCl, 5 mM MgCl_2_ were subjected to labelling with fluorescent dyes via maleimide chemistry, with a stochastic process occurring during derivatization with equimolar ratios of the dye molecules. A fivefold excess of a solution containing equimolar concentrations of the fluorescent FRET-pair dyes Atto520 (donor) and Atto647N (acceptor) were added and incubated at room temperature for 60 min. Subsequently, the protein was separated from unbound dye via size exclusion chromatography. Due to the stochastic labelling, the protein solution contained equimolar ratios of both dyes, with a total degree of labelling of approximately 0.2.

### Single-molecule measurements

FCS was performed as described previously^22^. Briefly, a 50 µL volume containing a highly diluted protein sample with concentrations in the low picomolar to high femtomolar range was applied on a coverslip on an inverted microscope (IX71, Olympus, Tokyo, Japan). When indicated, 1 mM ATP or AMP-PNP, respectively, was added to the sample. Using an optical setup with a confocal objective (UPlanSApo 60x water immersion, Olympus, Tokyo, Japan), an attenuated beam of a 491 nm laser was focussed into the sample and emitting light was passed through a pinhole and then split (via a dichroic mirror) into two lines, filtered with either a 535/50 BrightLine HC filter (Donor) or a 635 LP Edge Basic Longpass filter (AHF, Tuebingen, Germany) to collect the donor emission or the acceptor emission only and detected using two single photon avalanche diodes (Excelitas, Québec, Canada). The signals were correlated using a TCSPC (time-correlated single photon counting, DCP 230 card, Becker & Hickl, Germany) and the software burst analyzer (Becker & Hickl, Germany). A donor and acceptor fluorescence labelled double-stranded DNA molecule served as a control^22^. Events with a duration between 5 and 50 ms were automatically selected, with a maximum intensity of 60 counts per ms, when signals from both detectors, donor and acceptor, were observed.

### Simulation of protein conformations

All protein and ligand structures were prepared in Schrödinger Maestro^23^ software. For more details please refer to Yuan et al.^24^. The NMA has been done in ProDy3 tool^25^, a plugin of VMD^26^ software. NMA is a technique to investigate the vibrational motion of a harmonic oscillating system in the immediate vicinity of its equilibrium^27^. The motions studied are of small amplitude in a potential well and they cannot cross barriers of energy. NMA of proteins is based on the hypothesis that the vibrational normal modes exhibiting the lowest frequencies (also named soft modes) describe the largest movements in a protein and are the ones that are functionally relevant^28^. We performed NMA analysis using two crystal structures for the open (PDB: 1TF2) and the wide open (PDB: 1M74) states, respectively.

## Electronic supplementary material

Supplementary Information

Description of Additional Supplementary Information

Supplemental movie 1

Supplemental movie 2

## Data Availability

The datasets generated during and/or analysed during the current study are available from the corresponding author on reasonable request.
