# Supplementary material for: Large conformational changes of a highly dynamic pre-protein binding domain in SecA
Source: Commun Biol. 2018 Sep 3;1:130. doi: 10.1038/s42003-018-0133-4 (PMC6123708; doi:10.1038/s42003-018-0133-4)
Supplement: Supplementary file 1 — Supplementary Information [file 42003_2018_133_MOESM1_ESM.pdf]

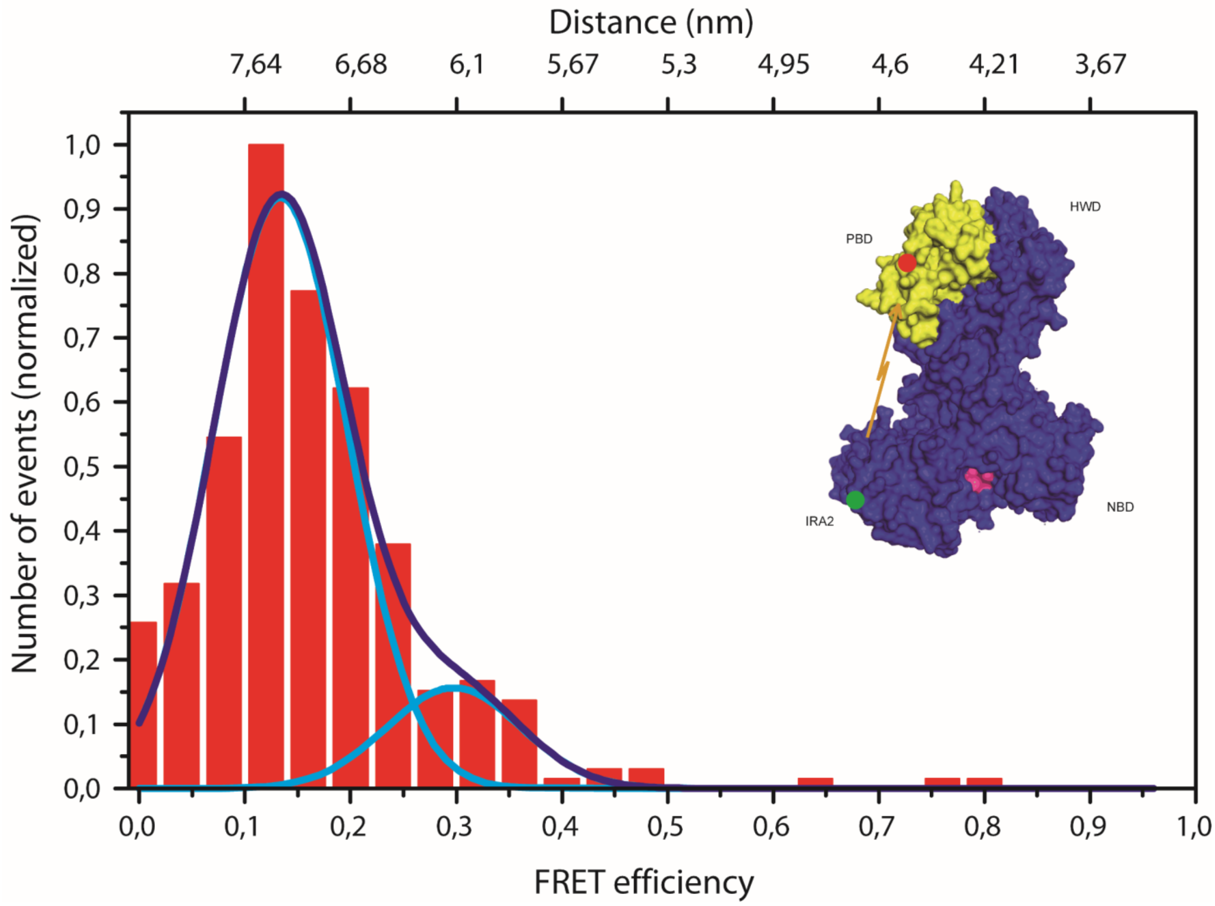

Supplementary Figure 1. After AMP-PNP binding the PBD moves away from the IRA2 domain. Histogram of the smFRET data obtained with SecA 329C-472C in the presence of AMP-PNP. The data were fitted with a bimodal Gaussian distribution and the distance between the fluorophores in the PBD and the IRA2 domain was determined with 7.3 nm indicating that the PDB moves towards the HWD.

Insets show the position of the fluorescent labels in SecA. To illustrate the movement of the PBD, the atomic SecA models 1TF2 (top) and 1M74 (bottom) are shown. The distance between the PBD at residue 329 (red dot) and the IRA2 domain at 472 (green dot) is high-lighted with arrows and is 5.1 nm in the *B. subtilis* open structure (1TF2) and 6.1 nm in the wide open structure (1M74) between the corresponding residues (D309 and K452).
