# Supplementary material for: Large conformational changes of a highly dynamic pre-protein binding domain in SecA
Source: Commun Biol. 2018 Sep 3;1:130. doi: 10.1038/s42003-018-0133-4 (PMC6123708; doi:10.1038/s42003-018-0133-4)
Supplement: Supplementary file 2 — Description of Additional Supplementary Information [file 42003_2018_133_MOESM2_ESM.docx]

**Description of Additional Supplementary Files**

File Name: Supplementary Movie 1

Description: The intrinsic movements of SecA in the open state. The length of the arrows is correlated with the flexibility of related motifs. Yellow region: the pre-protein binding domain.

File Name: Supplementary Movie 2

Description: The intrinsic movements of SecA in the wide open state. The length of the arrows is correlated with the flexibility of related motifs. Yellow region: the pre-protein binding domain.
